# Supplementary material for: Amelioration of age-related cognitive decline and anxiety in mice by Centella asiatica extract varies by sex, dose and mode of administration
Source: Front Aging. 2024 May 6;5:1357922. doi: 10.3389/fragi.2024.1357922 (PMC11102990; doi:10.3389/fragi.2024.1357922)
Supplement: Supplementary file 1 [file DataSheet1.docx]

Supplementary Material

**Supplementary Figure 1: Time immobile in the Open field (OF) test (A) and time in the dark in the Light-Dark Box (LD-Box) were not affected by age or treatment n=17-20 (6-8F, 11-12M) per condition**


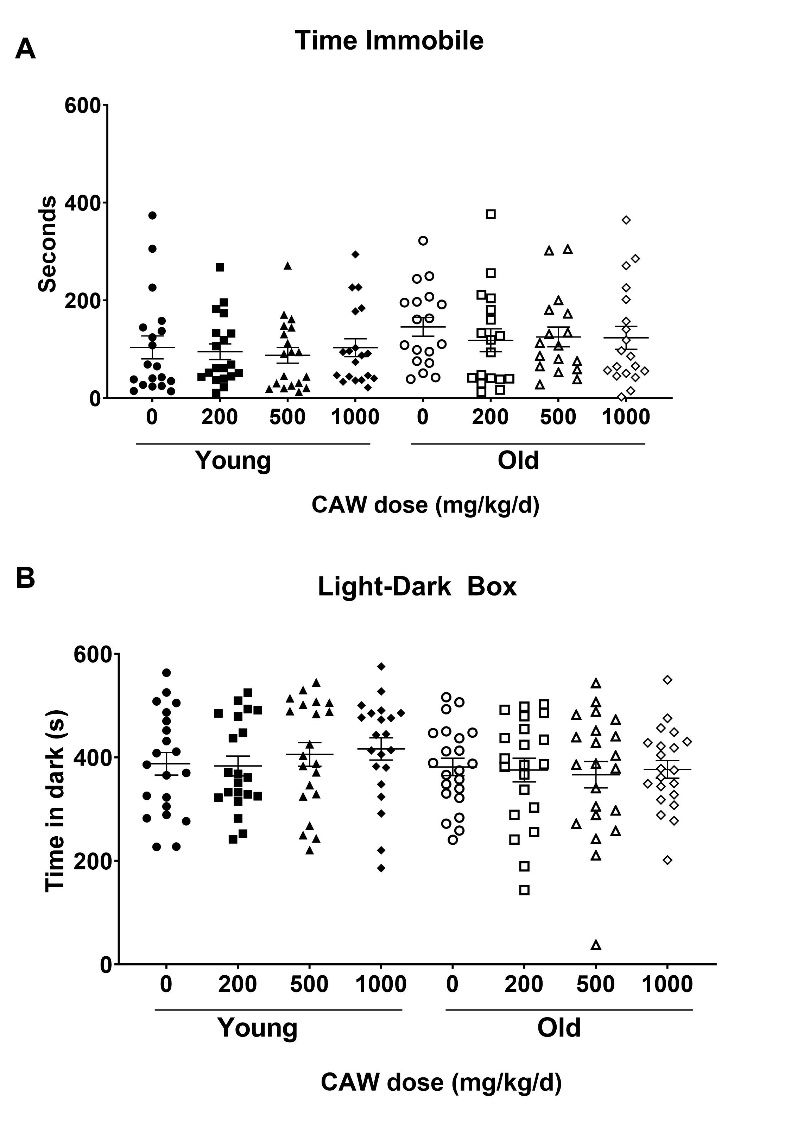


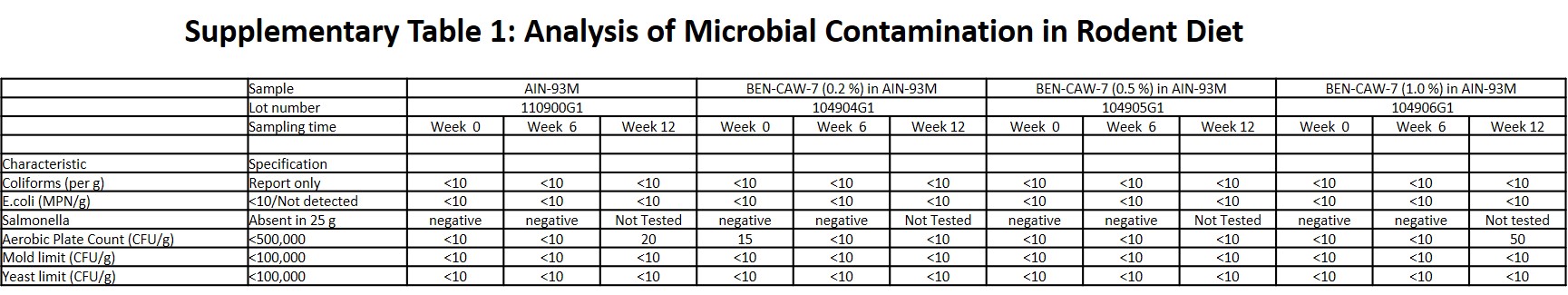


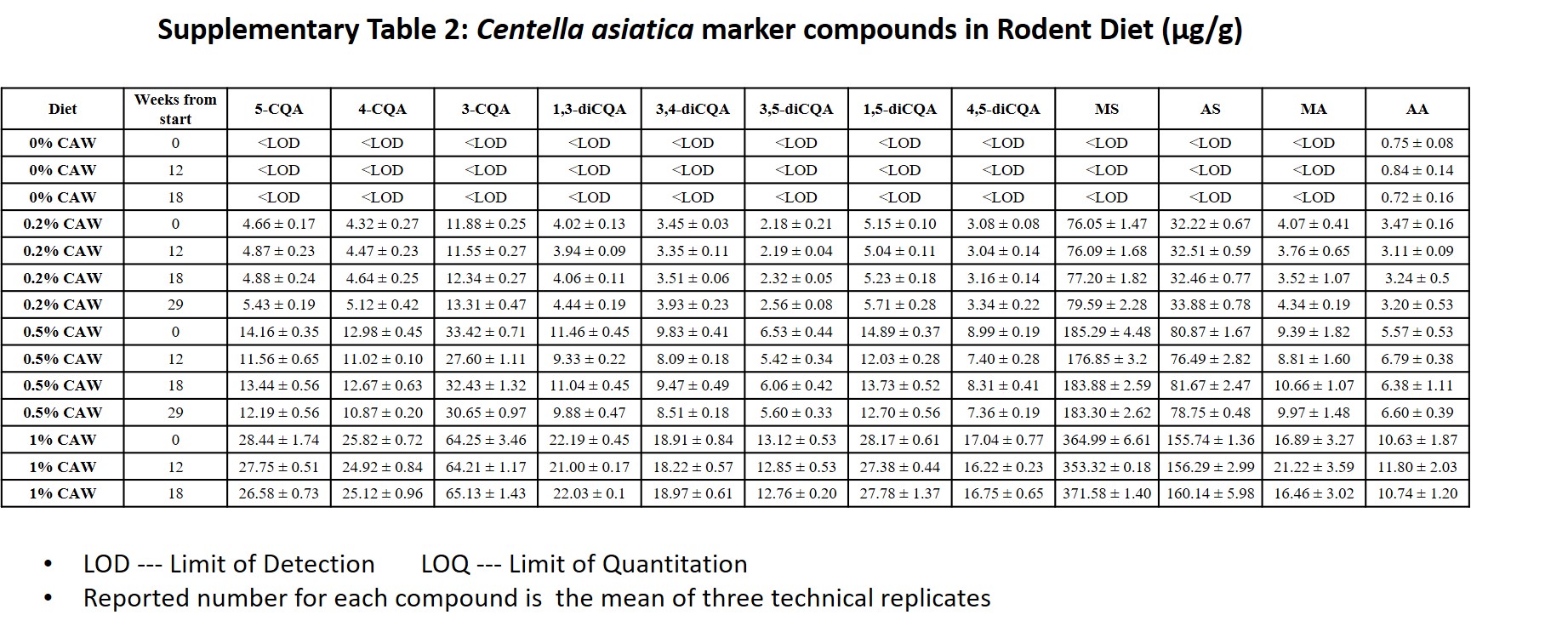


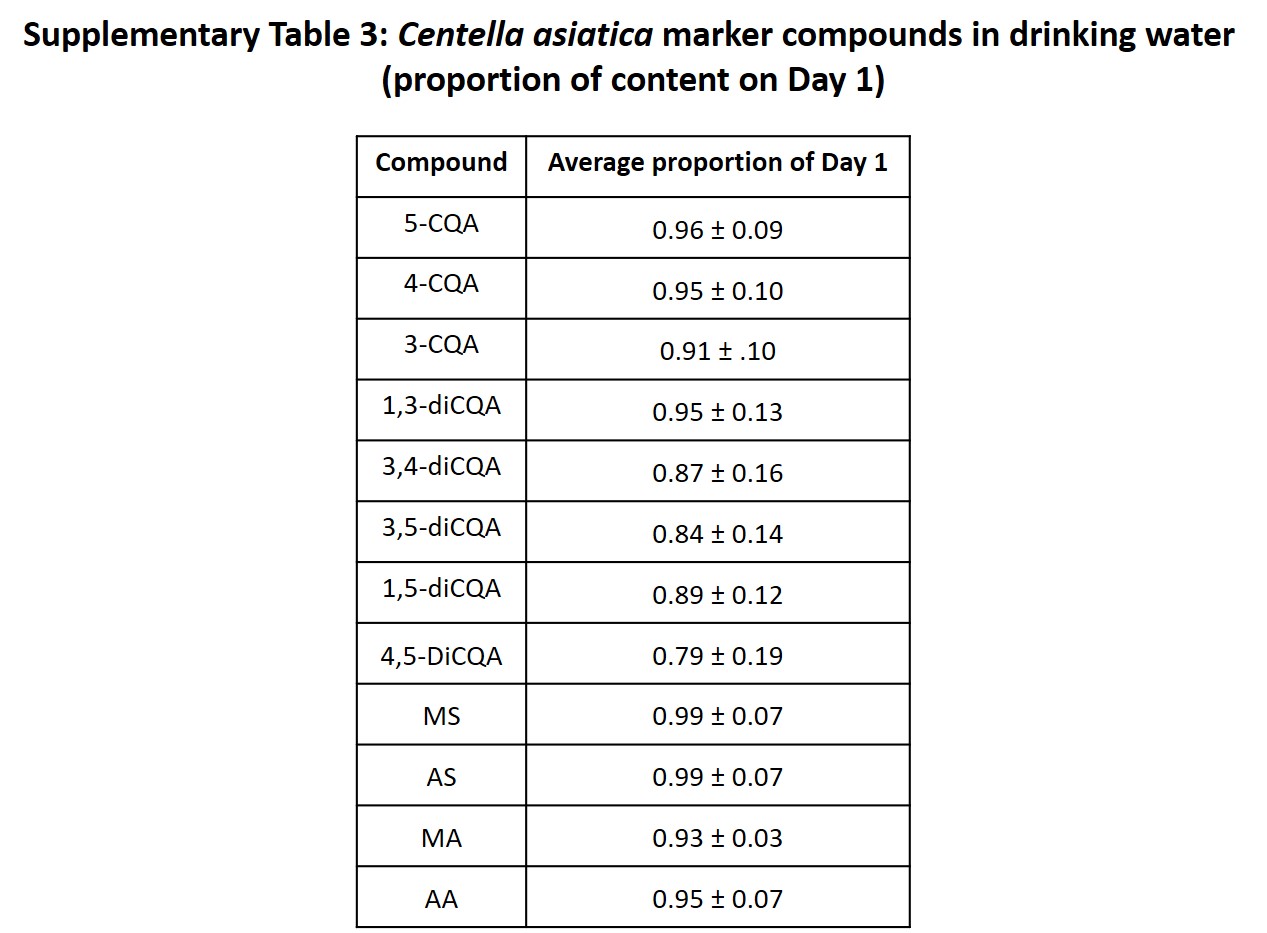


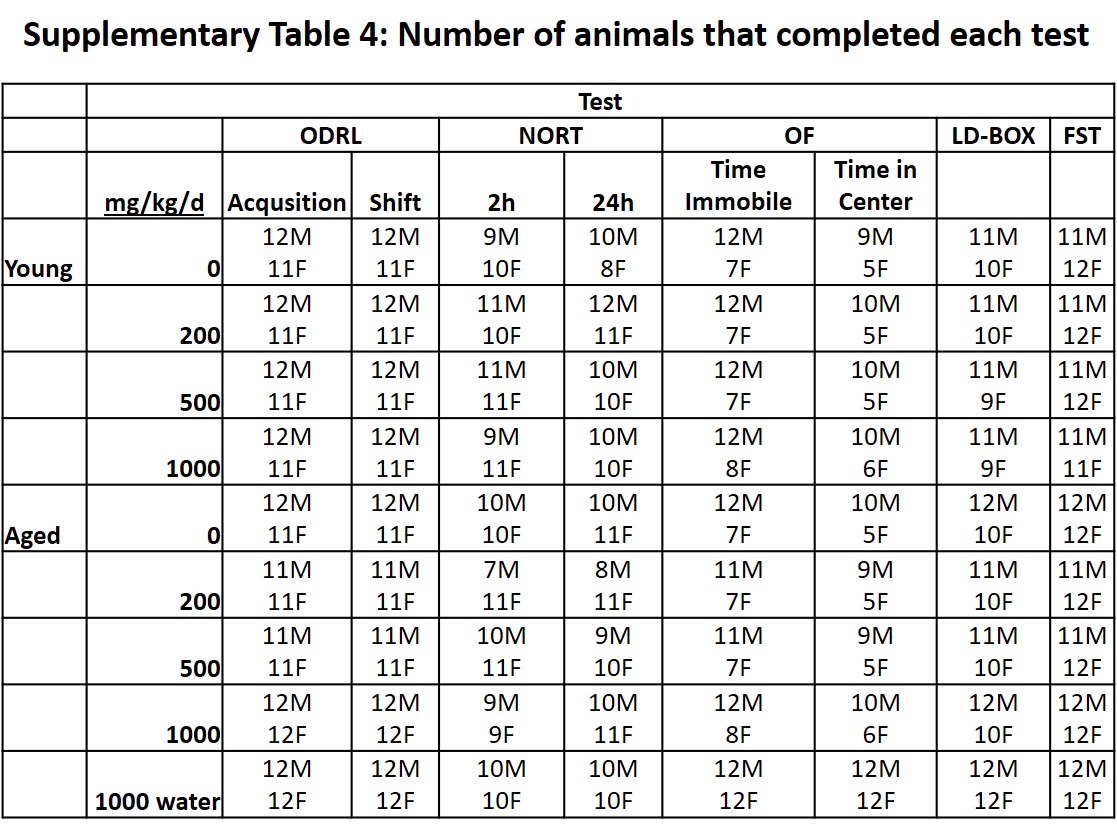


Although n=12 per group were intended for each test we were unable to obtain data from all animals in each test due to a variety of reasons including technical issues with the software, non-participation of mice in the task, and COVID-related issues with the experimenters


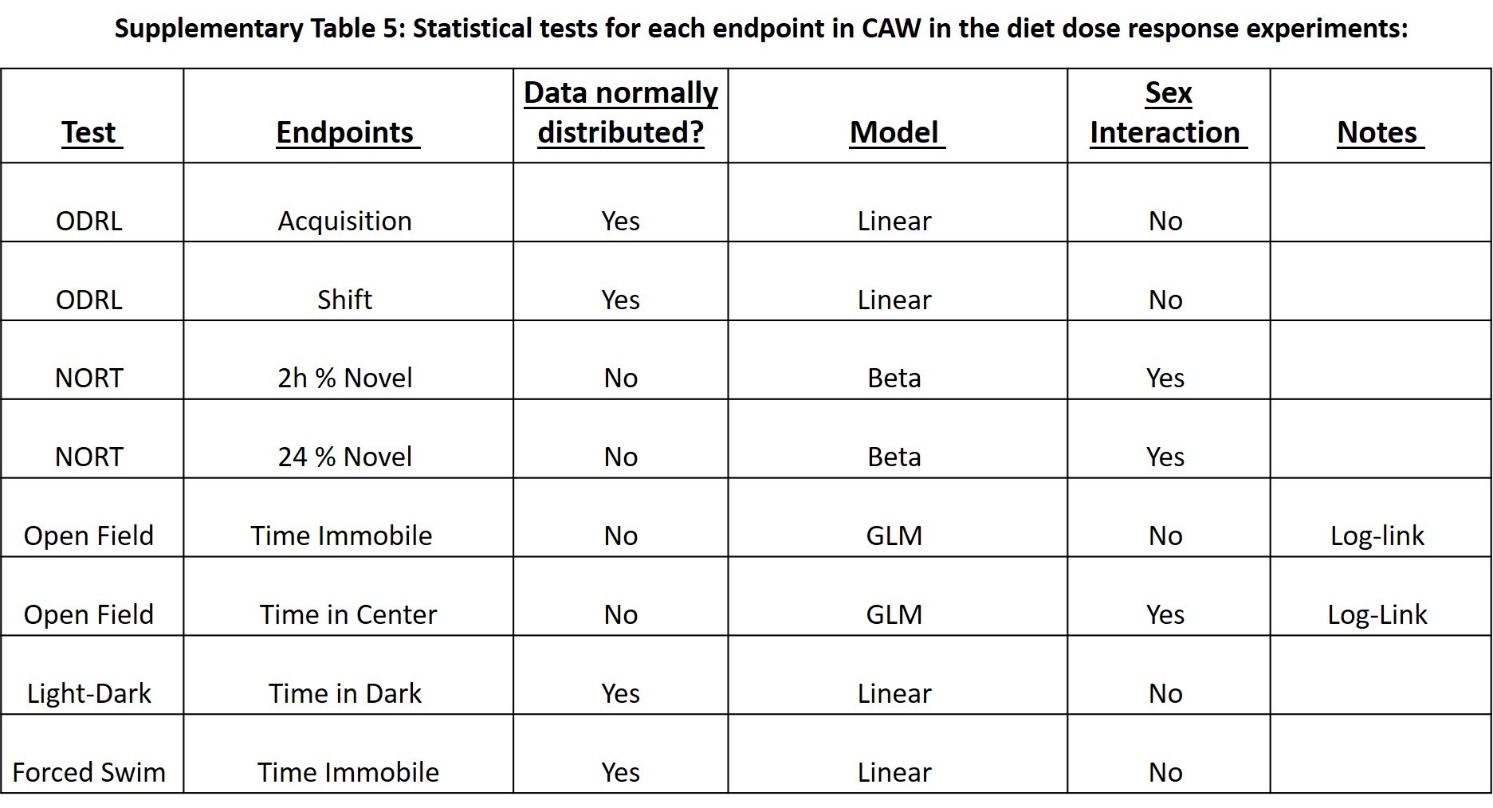


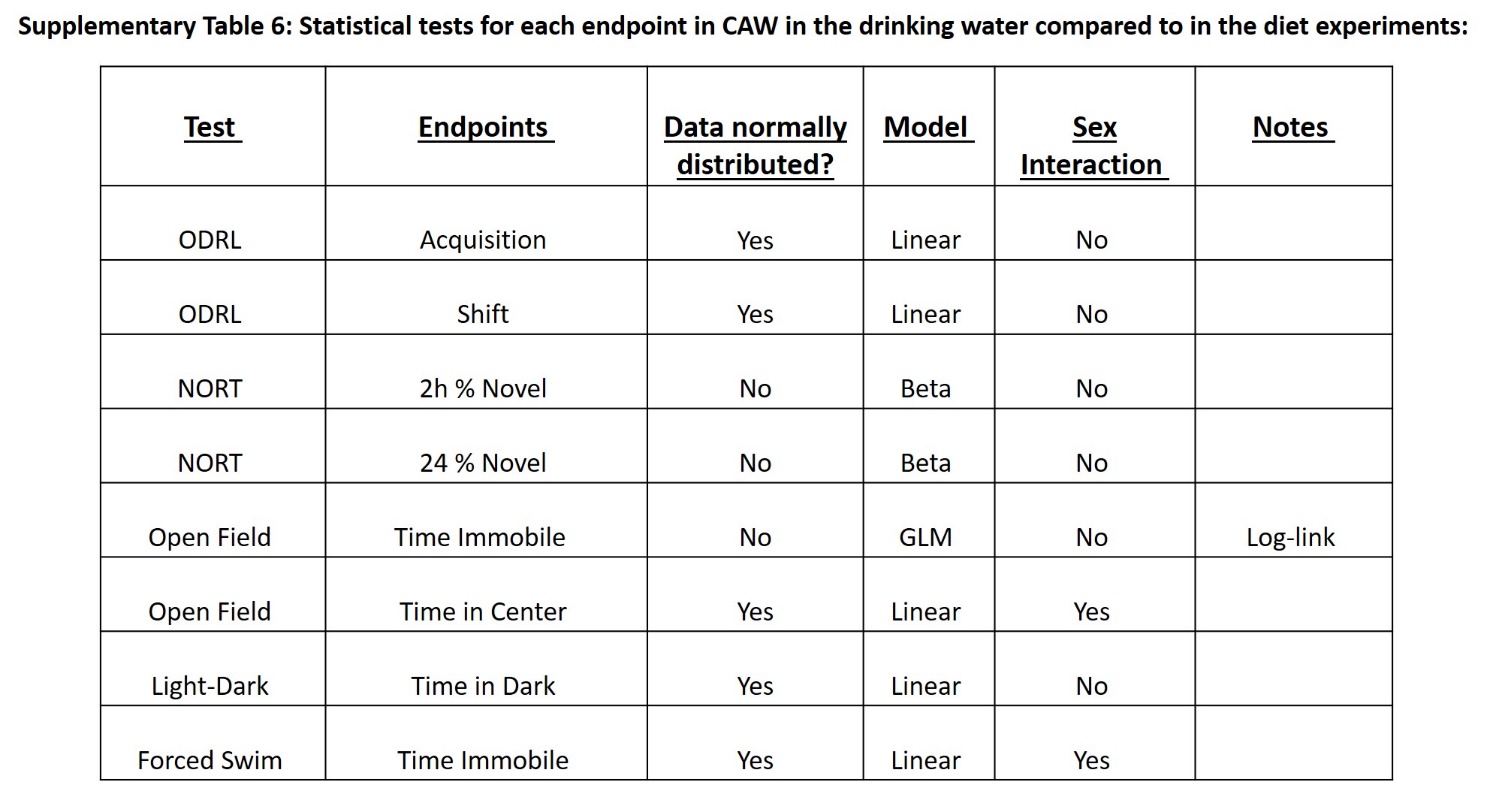


|  | **SUPPLEMENTARY TABLE 7: CAW ADMINISTERED IN THE DIET** | | | | |  |  |  |  |
| --- | --- | --- | --- | --- | --- | --- | --- | --- | --- |
| **ODRL - Acquisition** |  |  |  |  |  |  |  | |  |
| contrast | estimate | SE | df | lower.CL | upper.CL | t.ratio | p.value | |  |
| Young 0 vs Old 0 | -6.652173913 | 0.717327 | 173 | -8.553731583 | -4.750616243 | -9.27355 | 5.55E-16 | |  |
| Young 0 vs Young 200 | -0.173913043 | 0.625885 | 173 | -1.83306576 | 1.485239673 | -0.27787 | 0.999873 | |  |
| Young 0 vs Young 500 | 0.260869565 | 0.583385 | 173 | -1.285620915 | 1.807360045 | 0.447165 | 0.997453 | |  |
| Young 0 vs Young 1000 | 0.52173913 | 0.558715 | 173 | -0.959353478 | 2.002831739 | 0.93382 | 0.890765 | |  |
| Old 0 vs Old 200 | 2.739142322 | 0.80844 | 173 | 0.5960558 | 4.882228845 | 3.388184 | 0.005926 | |  |
| Old 0 vs Old 500 | 5.102772472 | 0.793902 | 173 | 2.998222838 | 7.207322106 | 6.427456 | 4.83E-09 | |  |
| Old 0 vs Old 1000 | 6.466408836 | 0.680213 | 173 | 4.663236907 | 8.269580764 | 9.506444 | 0 | |  |
|  |  |  |  |  |  |  |  | |  |
| **ODRL - Shift** |  |  |  |  |  |  |  | |  |
| contrast | estimate | SE | df | lower.CL | upper.CL | t.ratio | p.value | |  |
| Young 0 vs Old 0 | -5.869565217 | 0.718588 | 173 | -7.753762669 | -3.985367766 | -8.16819 | 1.63E-13 | |  |
| Young 0 vs Young 200 | -0.043478261 | 0.553032 | 173 | -1.493572291 | 1.40661577 | -0.07862 | 1 | |  |
| Young 0 vs Young 500 | 0.043478261 | 0.521746 | 173 | -1.324581286 | 1.411537808 | 0.083332 | 1 | |  |
| Young 0 vs Young 1000 | 0.086956522 | 0.547554 | 173 | -1.348775372 | 1.522688415 | 0.158809 | 0.999994 | |  |
| Old 0 vs Old 200 | 2.599525285 | 0.70951 | 173 | 0.739132056 | 4.459918513 | 3.663832 | 0.002063 | |  |
| Old 0 vs Old 500 | 4.990682522 | 0.687229 | 173 | 3.188712714 | 6.792652331 | 7.262041 | 2.65E-11 | |  |
| Old 0 vs Old 1000 | 5.615682522 | 0.658018 | 173 | 3.890306114 | 7.34105893 | 8.534242 | 1.60E-14 | |  |
|  |  |  |  |  |  |  |  | |  |
| **NORT 2h** |  |  |  |  |  |  |  | |  |
| sex | contrast | estimate | SE | asymp.LCL | asymp.UCL | z.ratio | p.value | |  |
| M | Young 0 vs Old 0 | 0.202453 | 0.111992 | -0.090375578 | 0.495281093 | 1.807739 | 0.306312 | |  |
| M | Young 0 vs Young 200 | -0.00953 | 0.093067 | -0.252874384 | 0.233815226 | -0.10239 | 1 | |  |
| M | Young 0 vs Young 500 | -0.05114 | 0.089486 | -0.285115303 | 0.182845031 | -0.57143 | 0.987068 | |  |
| M | Young 0 vs Young 1000 | -0.04017 | 0.094498 | -0.287257585 | 0.206916174 | -0.42509 | 0.997567 | |  |
| M | Old 0 vs Old 200 | -0.24029 | 0.106987 | -0.520033285 | 0.039447138 | -2.24601 | 0.124548 | |  |
| M | Old 0 vs Old 500 | -0.32645 | 0.100375 | -0.588905044 | -0.063999283 | -3.25232 | 0.006799 | |  |
| M | Old 0 vs Old 1000 | -0.2316 | 0.112079 | -0.524654777 | 0.061453839 | -2.06641 | 0.185169 | |  |
| F | Young 0 vs Old 0 | 0.072065 | 0.08578 | -0.154310395 | 0.298441108 | 0.840116 | 0.94099 | |  |
| F | Young 0 vs Young 200 | -0.13883 | 0.074239 | -0.334743575 | 0.057092857 | -1.86998 | 0.298006 | |  |
| F | Young 0 vs Young 500 | 0.230366 | 0.087423 | -0.00034616 | 0.461077828 | 2.635059 | 0.050591 | |  |
| F | Young 0 vs Young 1000 | 0.090309 | 0.086307 | -0.137456166 | 0.318074965 | 1.046375 | 0.850177 | |  |
| F | Old 0 vs Old 200 | 0.118084 | 0.088468 | -0.115385484 | 0.35155364 | 1.334761 | 0.661054 | |  |
| F | Old 0 vs Old 500 | 0.044315 | 0.095869 | -0.208685731 | 0.297316663 | 0.462248 | 0.997718 | |  |
| F | Old 0 vs Old 1000 | 0.042838 | 0.087783 | -0.188821975 | 0.274497763 | 0.488 | 0.996833 | |  |
|  |  |  |  |  |  |  |  | |  |
| **NORT 24h** |  |  |  |  |  |  |  | |  |
| sex | contrast | estimate | SE | asymp.LCL | asymp.UCL | z.ratio | p.value | |  |
| M | Young 0 vs Old 0 | -0.02421 | 0.080981 | -0.238190498 | 0.189765797 | -0.29899 | 0.999867 | |  |
| M | Young 0 vs Young 200 | 0.128883 | 0.084035 | -0.093164992 | 0.35093041 | 1.533675 | 0.520681 | |  |
| M | Young 0 vs Young 500 | 0.041902 | 0.084999 | -0.182693668 | 0.266496884 | 0.492964 | 0.99682 | |  |
| M | Young 0 vs Young 1000 | -0.09842 | 0.074852 | -0.296204301 | 0.099362963 | -1.31486 | 0.680471 | |  |
| M | Old 0 vs Old 200 | 0.249303 | 0.09401 | 0.000898677 | 0.497706775 | 2.651876 | 0.049221 | |  |
| M | Old 0 vs Old 500 | 0.125546 | 0.088643 | -0.10867651 | 0.359767866 | 1.416311 | 0.606355 | |  |
| M | Old 0 vs Old 1000 | -0.04628 | 0.075598 | -0.246029773 | 0.153479755 | -0.61212 | 0.988847 | |  |
| F | Young 0 vs Old 0 | 0.111492 | 0.096844 | -0.143184873 | 0.366168665 | 1.151252 | 0.77502 | |  |
| F | Young 0 vs Young 200 | 0.000631 | 0.093712 | -0.24580807 | 0.247070976 | 0.006738 | 1 | |  |
| F | Young 0 vs Young 500 | 0.061367 | 0.097942 | -0.196197534 | 0.318930611 | 0.62656 | 0.984739 | |  |
| F | Young 0 vs Young 1000 | 0.090469 | 0.09866 | -0.168984851 | 0.349921897 | 0.916969 | 0.904586 | |  |
| F | Old 0 vs Old 200 | 0.001847 | 0.09258 | -0.241616434 | 0.245311051 | 0.019954 | 1 | |  |
| F | Old 0 vs Old 500 | -0.23063 | 0.083645 | -0.450598105 | -0.01066532 | -2.75727 | 0.035067 | |  |
| F | Old 0 vs Old 1000 | -0.04816 | 0.091615 | -0.289089704 | 0.19276389 | -0.52571 | 0.994263 | |  |
|  |  |  |  |  |  |  |  | |  |
| **Open Field - time in center** |  |  |  |  |  |  |  | |  |
| contrast | ratio | SE | asymp.LCL | asymp.UCL | z.ratio | p.value | sex | | |
| Young 0 vs Old 0 | 0.761995409 | 0.1191964 | 0.505248406 | 1.149210955 | -1.737649708 | 0.36105138 | Male | | |
| Young 0 vs Young 200 | 0.867376203 | 0.097414542 | 0.645784853 | 1.165003288 | -1.266879018 | 0.687493898 | Male | | |
| Young 0 vs Young 500 | 0.962007422 | 0.137014131 | 0.661764526 | 1.39847067 | -0.271954011 | 0.999888599 | Male | | |
| Young 0 vs Young 1000 | 0.891290111 | 0.134365225 | 0.599850916 | 1.324325831 | -0.763399845 | 0.954606368 | Male | | |
| Old 0 vs Old 200 | 0.902708986 | 0.160521641 | 0.565839127 | 1.440132849 | -0.575603544 | 0.989171322 | Male | | |
| Old 0 vs Old 500 | 0.809255293 | 0.115232272 | 0.556733563 | 1.176315159 | -1.486315176 | 0.529138411 | Male | | |
| Old 0 vs Old 1000 | 1.006408155 | 0.163043429 | 0.65760153 | 1.54022965 | 0.039429025 | 1 | Male | | |
| Young 0 vs Old 0 | 1.82670467 | 0.264423147 | 1.244577348 | 2.681110946 | 4.162322587 | 0.00034607 | Female | | |
| Young 0 vs Young 200 | 1.686831876 | 0.344262016 | 0.982015015 | 2.897513514 | 2.561896507 | 0.064158492 | Female | | |
| Young 0 vs Young 500 | 1.834103817 | 0.247783304 | 1.28201857 | 2.623937665 | 4.489756244 | 5.02254E-05 | Female | | |
| Young 0 vs Young 1000 | 1.799870382 | 0.26788615 | 1.213096182 | 2.670467058 | 3.94873045 | 0.000760731 | Female | | |
| Old 0 vs Old 200 | 0.823850294 | 0.112379838 | 0.573865614 | 1.182732143 | -1.420490964 | 0.627090312 | Female | | |
| Old 0 vs Old 500 | 0.974572649 | 0.231569309 | 0.51911954 | 1.829620688 | -0.108396489 | 0.999999901 | Female | | |
| Old 0 vs Old 1000 | 1.263259936 | 0.231886702 | 0.776549421 | 2.055021385 | 1.273114524 | 0.733371376 | Female | | |
|  |  |  |  |  |  |  |  | |  |
| **Forced Swim - time immobile** |  |  |  |  |  |  |  | |  |
| contrast | ratio | SE | asymp.LCL | asymp.UCL | null | z.ratio | p.value | |  |
| Young 0 vs Old 0 | 1.351671485 | 0.192178 | 0.930901 | 1.962630863 | 1 | 2.11947 | 0.174151 | |  |
| Young 0 vs Young 200 | 0.988209972 | 0.125776 | 0.707713 | 1.379879406 | 1 | -0.09318 | 1 | |  |
| Young 0 vs Young 500 | 1.007104505 | 0.102098 | 0.771944 | 1.313903198 | 1 | 0.069831 | 1 | |  |
| Young 0 vs Young 1000 | 0.929251125 | 0.104458 | 0.691953 | 1.247928458 | 1 | -0.65275 | 0.979914 | |  |
| Old 0 vs Old 200 | 1.016651718 | 0.155353 | 0.680917 | 1.517924154 | 1 | 0.108074 | 1 | |  |
| Old 0 vs Old 500 | 1.107486912 | 0.185568 | 0.713607 | 1.718771739 | 1 | 0.609303 | 0.986048 | |  |
| Old 0 vs Old 1000 | 0.977463055 | 0.149046 | 0.65523 | 1.458165984 | 1 | -0.14949 | 0.999998 | |  |

|  | **SUPPLEMENTARY TABLE 8: CAW IN THE WATER VS.DIET** | | | | |  |  |
| --- | --- | --- | --- | --- | --- | --- | --- |
| **ODRL- Acquisition** |  |  |  |  |  |  |  |
| contrast | estimate | SE | df | lower.CL | upper.CL | t.ratio | p.value |
| Young 0 vs Old 0 | -6.65217 | 0.718704 | 89 | -8.528067541 | -4.77628 | -9.2558 | 5.52E-14 |
| Young 0 vs Old 1000 CAW chow | -0.18164 | 0.594932 | 89 | -1.734478609 | 1.371194 | -0.30532 | 0.989859 |
| Young 0 vs Old 1000 CAW water | 1.151691 | 0.549853 | 89 | -0.283483625 | 2.586865 | 2.094544 | 0.159946 |
| Old 0 vs Old 1000 CAW chow | 6.470531 | 0.68469 | 89 | 4.683417628 | 8.257645 | 9.450311 | 6.66E-15 |
| Old 0 vs Old 1000 CAW water | 7.803865 | 0.645906 | 89 | 6.117981765 | 9.489748 | 12.08205 | 0 |
| Old 1000 CAW chow vs Old 1000 CAW water | 1.333333 | 0.499679 | 89 | 0.029117305 | 2.637549 | 2.668379 | 0.043078 |
|  |  |  |  |  |  |  |  |
| **ODRL - Shift** |  |  |  |  |  |  |  |
| contrast | estimate | SE | df | lower.CL | upper.CL | t.ratio | p.value |
| Young 0 vs Old 0 | -5.86957 | 0.719703 | 89 | -7.736671885 | -4.00246 | -8.15554 | 2.96E-12 |
| Young 0 vs Old 1000 CAW chow | -0.25052 | 0.458005 | 89 | -1.438714253 | 0.937668 | -0.54699 | 0.944859 |
| Young 0 vs Old 1000 CAW water | 0.83281 | 0.483888 | 89 | -0.422526418 | 2.088146 | 1.721082 | 0.308243 |
| Old 0 vs Old 1000 CAW chow | 5.619042 | 0.662291 | 89 | 3.900878334 | 7.337205 | 8.48425 | 6.64E-13 |
| Old 0 vs Old 1000 CAW water | 6.702375 | 0.680447 | 89 | 4.937110975 | 8.467639 | 9.849966 | 1.11E-15 |
| Old 1000 CAW chow vs Old 1000 CAW water | 1.083333 | 0.384681 | 89 | 0.085364589 | 2.081302 | 2.816183 | 0.02827 |
|  |  |  |  |  |  |  |  |
| **NORT 2h** |  |  |  |  |  |  |  |
| contrast | estimate | SE | asymp.LCL | asymp.UCL | z.ratio | p.value |  |
| Young 0 vs Old 0 | 0.13245 | 0.063759 | -0.031221434 | 0.296122339 | 2.077364 | 0.159917 |  |
| Young 0 vs Old 1000 CAW chow | 0.045142 | 0.061312 | -0.11224736 | 0.202531782 | 0.736275 | 0.882079 |  |
| Young 0 vs Old 1000 CAW water | -0.0545 | 0.055961 | -0.198159047 | 0.089151727 | -0.97395 | 0.763601 |  |
| Old 0 vs Old 1000 CAW chow | -0.08731 | 0.065312 | -0.254966298 | 0.080349815 | -1.33679 | 0.538335 |  |
| Old 0 vs Old 1000 CAW water | -0.18695 | 0.060704 | -0.342784245 | -0.031123981 | -3.07976 | 0.011341 |  |
| Old 1000 CAW chow vs Old 1000 CAW water | -0.09965 | 0.05805 | -0.248662395 | 0.049370653 | -1.71656 | 0.314021 |  |
|  |  |  |  |  |  |  |  |
| **NORT 24h** |  |  |  |  |  |  |  |
| contrast | estimate | SE | asymp.LCL | asymp.UCL | z.ratio | p.value |  |
| Young 0 vs Old 0 | 0.040447 | 0.060942 | -0.11590473 | 0.196798098 | 0.663695 | 0.91052 |  |
| Young 0 vs Old 1000 CAW chow | -0.00898 | 0.059369 | -0.161291733 | 0.143339342 | -0.15119 | 0.998766 |  |
| Young 0 vs Old 1000 CAW water | -0.0807 | 0.056832 | -0.226507721 | 0.065108154 | -1.41997 | 0.486121 |  |
| Old 0 vs Old 1000 CAW chow | -0.04942 | 0.058122 | -0.198539911 | 0.099694152 | -0.85033 | 0.829929 |  |
| Old 0 vs Old 1000 CAW water | -0.12115 | 0.05565 | -0.263921082 | 0.021628146 | -2.17694 | 0.129213 |  |
| Old 1000 CAW chow vs Old 1000 CAW water | -0.07172 | 0.053884 | -0.209967943 | 0.066520766 | -1.33107 | 0.542389 |  |
|  |  |  |  |  |  |  |  |
| **Open Field - Time in the Center** |  |  |  |  |  |  |  |
| contrast | estimate | SE | lower.CL | upper.CL | t.ratio | p.value | sex |
| Young 0 vs Old 0 | -42.51 | 25.57722 | -111.1247522 | 26.10475216 | -1.66203 | 0.357028 | Male |
| Young 0 vs Old 1000 CAW chow | -41.3727 | 24.98916 | -108.4099179 | 25.66446338 | -1.65563 | 0.360359 | Male |
| Young 0 vs Old 1000 CAW water | -33.45 | 24.48833 | -99.14362371 | 32.24362371 | -1.36596 | 0.527574 | Male |
| Old 0 vs Old 1000 CAW chow | 1.137273 | 24.98916 | -65.89991793 | 68.17446338 | 0.045511 | 0.999965 | Male |
| Old 0 vs Old 1000 CAW water | 9.06 | 24.48833 | -56.63362371 | 74.75362371 | 0.369972 | 0.98244 | Male |
| Old 1000 CAW chow vs Old 1000 CAW water | 7.922727 | 23.87346 | -56.12142089 | 71.96687543 | 0.331863 | 0.987191 | Male |
| Young 0 vs Old 0 | 125.6867 | 30.62253 | 40.31112754 | 211.0622058 | 4.104385 | 0.002679 | Female |
| Young 0 vs Old 1000 CAW chow | 157.37 | 28.12859 | 78.94755974 | 235.7924403 | 5.594663 | 8.69E-05 | Female |
| Young 0 vs Old 1000 CAW water | -231.888 | 22.31982 | -294.115916 | -169.6607506 | -10.3893 | 1.64E-10 | Female |
| Old 0 vs Old 1000 CAW chow | 31.68333 | 32.02581 | -57.6045427 | 120.9712094 | 0.989306 | 0.752324 | Female |
| Old 0 vs Old 1000 CAW water | -357.575 | 27.06675 | -433.0370283 | -282.1129717 | -13.2109 | 4.16E-12 | Female |
| Old 1000 CAW chow vs Old 1000 CAW water | -389.258 | 24.20924 | -456.7536234 | -321.7630433 | -16.0789 | 0 | Female |
|  |  |  |  |  |  |  |  |
| **Light Dark Box - Time in the Dark** |  |  |  |  |  |  |  |
| contrast | estimate | SE | lower.CL | upper.CL | t.ratio | p.value |  |
| Young 0 vs Old 0 | 6.293539 | 26.93906 | -64.05223385 | 76.63931236 | 0.233621 | 0.995372 |  |
| Young 0 vs Old 1000 CAW chow | 3.404903 | 27.54233 | -68.51617425 | 75.32598003 | 0.123624 | 0.999303 |  |
| Young 0 vs Old 1000 CAW water | -67.7626 | 24.22822 | -131.0296263 | -4.495660049 | -2.79685 | 0.030977 |  |
| Old 0 vs Old 1000 CAW chow | -2.88864 | 24.14467 | -65.93743544 | 60.16016272 | -0.11964 | 0.999368 |  |
| Old 0 vs Old 1000 CAW water | -74.0562 | 20.00023 | -126.2826459 | -21.829719 | -3.70277 | 0.00222 |  |
| Old 1000 CAW chow vs Old 1000 CAW water | -71.1675 | 20.8208 | -125.5367508 | -16.7983413 | -3.4181 | 0.005075 |  |
|  |  |  |  |  |  |  |  |
| **Forced Swim Test - Time Immobile** |  |  |  |  |  |  |  |
| contrast | estimate | SE | df | lower.CL | upper.CL | t.ratio | p.value |
| Young 0 vs Old 0 | 8.534848 | 28.10475 | 43 | -66.55256158 | 83.62226 | 0.30368 | 0.990137 |
| Young 0 vs Old 1000 CAW chow | -5.27348 | 28.10475 | 43 | -80.36089491 | 69.81393 | -0.18764 | 0.997619 |
| Young 0 vs Old 1000 CAW water | -0.70682 | 28.10475 | 43 | -75.79422825 | 74.38059 | -0.02515 | 0.999994 |
| Old 0 vs Old 1000 CAW chow | -13.8083 | 27.48699 | 43 | -87.2452691 | 59.6286 | -0.50236 | 0.958076 |
| Old 0 vs Old 1000 CAW water | -9.24167 | 27.48699 | 43 | -82.67860244 | 64.19527 | -0.33622 | 0.986721 |
| Old 1000 CAW chow vs Old 1000 CAW water | 4.566667 | 27.48699 | 43 | -68.8702691 | 78.0036 | 0.166139 | 0.998342 |
| Young 0 vs Old 0 | 91.21667 | 23.17415 | 44 | 29.38016405 | 153.0532 | 3.936139 | 0.0016 |
| Young 0 vs Old 1000 CAW chow | 90.10833 | 23.17415 | 44 | 28.27183072 | 151.9448 | 3.888313 | 0.001869 |
| Young 0 vs Old 1000 CAW water | 98.56667 | 23.17415 | 44 | 36.73016405 | 160.4032 | 4.253303 | 0.000618 |
| Old 0 vs Old 1000 CAW chow | -1.10833 | 23.17415 | 44 | -62.94483595 | 60.72817 | -0.04783 | 0.99996 |
| Old 0 vs Old 1000 CAW water | 7.35 | 23.17415 | 44 | -54.48650262 | 69.1865 | 0.317164 | 0.988804 |
| Old 1000 CAW chow vs Old 1000 CAW water | 8.458333 | 23.17415 | 44 | -53.37816928 | 70.29484 | 0.36499 | 0.983154 |
